# Homotopic Approximate Solutions for the Perturbed CKdV Equation with Variable Coefficients

**DOI:** 10.1155/2014/593642

**Published:** 2014-03-05

**Authors:** Dianchen Lu, Tingting Chen, Baojian Hong

**Affiliations:** Center of Nonlinear Science Research, Jiangsu University, Zhenjiang, Jiangsu 212013, China

## Abstract

This work concerns how to find the double periodic form of approximate solutions of the perturbed combined KdV (CKdV) equation with variable coefficients by using the homotopic mapping method. The obtained solutions may degenerate into the approximate solutions of hyperbolic function form and the approximate solutions of trigonometric function form in the limit cases. Moreover, the first order approximate solutions and the second order approximate solutions of the variable coefficients CKdV equation in perturbation *εu* ^*n*^ are also induced.

## 1. Introduction

To solve the nonlinear partial differential equation (NPDE) has been an attractive research topic for mathematicians and physicists. Nonlinear evolution equations with variable coefficients can describe the physical phenomenon more accurately, and it is of great significance to study how to find the solutions of nonlinear evolution equations with variable coefficients.

In recent years, many researchers have developed various approaches for attaining the exact solutions and approximate of NPDE, such as the inverse scattering method [1], homogeneous balance method [2], elliptic function method [3], and perturbation method [4]. A recently reported analytic approximate method, the homotopic mapping method proposed in [5], has been applied to solve many nonlinear problems in engineering and technology effectively, like the nonlinear vibration of [6], boundary layer flow of [7], and so on [8–12]. However, the above works only studied the soliton approximate solutions of equations with constant coefficients. In this work, we applied the homotopic mapping method to the variable coefficients perturbed CKdV equation and obtained the approximate solution of the Jacobi elliptic function form.

## 2. Model and Homotopy Mapping

In this work, we focus on the perturbed CKdV equation with variable coefficients

(1) ut+a(t)uux+b(t)u2ux+c(t)u3x=f(x,t,u,ux,ut),

where *a*(*t*), *b*(*t*), *c*(*t*) is any function about *t*, *f* is a disturbance term, and *f* is the sufficiently smooth function. This equation is widely used in the field of plasma physics [13], fluid mechanics [14], and quantum field theory [15]. It is fascinating to observe that when *a*(*t*), *b*(*t*), *c*(*t*) is constant, *f* = 0, (1) becomes the well-known combined KdV equation, the equation in plasma physics which describes the acoustic wave propagation of a small-amplitude ion without Landau decay. It can be used as a model equation in fluid mechanics; related research can be referred to in [16, 17].

When *f* = *R*(*t*), (1) becomes the forced combined KdV equation, and the exact solutions of various forms are given in [18, 19], such as solitary wave solutions, trigonometric function solutions, and Jacobian elliptic function solutions. When *b*(*t*) = 0, [20] studied the elliptic function solution of composite form.

Next, we study the approximate solution of (1). In order to simplify (1), set

(2) x′=c3a(t)c1c(t)x,  t′=∫0tc3a3(τ)c13c(τ)dτ,tt′=c3a3(t)c13c(t),

where *c*_1_, *c*_3_ are any constants.

By setting *c*_2_(*t*) = *b*(*t*)*c*_1_/*a*(*t*), (1) can be expressed as

(3) ut′+c1uux′+c2(t)u2ux′+c3u3x′=f∗(x′,t′,u,ux′,ut′),

where

(4) f∗(x′,t′,u,ux′,ut′)  =(f[c1c(t)c3a(t)x′,t(t′),u,c3a(t)c1c(t)ux′,    b(t)c2c3a3(t)c13c(t)ut′])×(tt′)−1.

For the sake of convenience, let = *x*′, *t* = *t*′, *f*(*x*, *t*, *u*, *u*_*x*_, *u*_*t*_) = f*(*x*′, *t*′, *u*, *u*_*x*′_, *u*_*t*′_), and (3) can be written as

(5) ut+c1uux+c3u3x=−c2(t)u2ux+f(x,t,u,ux,ut);

the study on the solution of (1) is translated into the solution of (5).

In order to get the solution of (5), we lead in homotopy mapping.

### 2.1. Introduction of Homotopy Mapping

Assume we are given nonlinear equation *A*(*u*) − *f*(*r*) = 0, *r* ∈ *Ω* and boundary condition *B*(*u*, ∂*u*/∂*n*) = 0, *r* ∈ Γ, where *A* is the general differential operator, *B* is the boundary operator, *f*(*r*) is the known analytic function, and Γ is the boundary of the region *Ω*. Generally speaking, the operator *A* can be decomposed into linear part *L* and nonlinear part *N*. So equation *A*(*u*) − *f*(*r*) = 0 can be written as *L*(*u*) + *N*(*u*) − *f*(*r*) = 0.

Now we set up homotopy mapping: *H*(*u*, *p*) : *Ω* × [0,1] → *R*,

(6) H(u,p)=L(u)−L(v)+p(L(v)+N(u)−f(r)),

where *p* is parameter, *v* is auxiliary function, and *L*(*v*) + *N*(*v*) = 0.

By (6), we obtain

(7) H(u,0)=L(u)−L(v),H(u,1)=A(u)−f(r)=0.

As can be seen from 0 to 1 of *p* is the process of *L*(*u*) − *L*(*v*) to *A*(*u*) − *f*(*r*) of *H*(*u*, *p*); this is the homotopy deformation. Set

(8) $$\begin{array}{c} \tilde{u}\left(x,t,p\right)=\overset{\infty }{\underset{i=0}{\sum }}u_{i}\left(x,t\right)p^{i}=u_{0}+pu_{1}+p^{2}u_{2}+\cdots \end{array}$$

is the solution of *H*(*u*, *p*) = 0. So when *p* = 0, $\tilde{u}\left(x,t,0\right)=u_{0}\left(x,t\right)$ is the solution of *L*(*u*) − *L*(*v*) = 0; when *p* → 1, the approximate solution of *A*(*u*) − *f*(*r*) = 0 is *u*(*x*, *t*) = *u*_0_ + *u*_1_ + *u*_2_ + ⋯.

### 2.2. Approximate Solution of the Jacobi Elliptic Function Form

Aiming at (5), we set up homotopy mapping *H*(*u*, *p*)  :  *R* × *I* → *R*,

(9) H(u,p)=L(u)−L(v)+p(L(v)+c1uux  +c2(t)u2ux−f(x,t,u,ux,ut)),

where *R* = (−*∞*, +*∞*), *I* = [0,1], *v* is the auxiliary function; linear operator *L* is expressed as *L*(*u*) = *u*_*t*_ + *c*_3_ *u*_3*x*_.

By using the generalized ellipse method [21], we can get that the typical KdV equation corresponding to (5)

(10) vt+c1vvx+c3v3x=0

has the following elliptic function solution:

(11) v1(x,t)=c0−6k2c3m2sn(ξ,m)+12k2c3(m2−1)cn(ξ,m)c1(sn(ξ,m)+cn(ξ,m)+dn(ξ,m))+3k2c3m4sn2(ξ,m)−12k2c3cn2(ξ,m)c1(sn(ξ,m)+cn(ξ,m)+dn(ξ,m))2.

When *m* → 1, *v*_1_(*x*, *t*) degenerates to the following solitary wave solution:

(12) v1.1(x,t)=c0+−6k2c3tanhξc1(tanhξ+2 sec hξ)+3k2c3tanh2ξ−12k2c3sec h2ξc1(tanhξ+2 sec hξ)2.

When *m* → 0, *v*_1_(*x*, *t*) degenerates to the trigonometric function solution

(13) v1.2(x,t)=c0+12k2c3cos⁡ξc1(sinξ+cos⁡ξ+1)−12k2c3cos⁡2ξc1(sinξ+cos⁡ξ+1)2,

where *ξ* = *kx* + [−*c*_0_ *kc*_1_ + *k* ^3^ *c*_3_(4*m* ^2^ − 5)]*t* + *ξ*_0_, *k*, *c*_0_, *ξ*_0_ is any constant, *m* is the module, and 0 ≤ *m* ≤ 1.

One can easily prove that *H*(*u*, 1) = 0 and (5) is the same, so the solution *u*(*x*, *t*) of (5) is the solution of *H*(*u*, *p*) = 0 when under the condition *p* → 1.

Let

(14) u~(x,t,p)=∑i=0∞ui(x,t)pi=u0+pu1+p2u2+⋯

be the solution of *H*(*u*, *p*) = 0; by [22] we can know this series is uniformly convergent in the *p* ∈ [0,1]. Thus, it yields that

(15) u=∑i=0∞ui(x,t)=u0+u1+u2+⋯.

## 3. Approximate Solution 

In order to obtain the approximate solution of (5), we substitute (14) into equation *H*(*u*, *p*) = 0. By taking the auxiliary function *v* = *v*_1_(*x*, *t*) and comparing the coefficients of the same power of *p*, one can obtain that

(16) p0:L(u0)=L(v)=L(v1),

(17) p1:L(u1)=−L(v1)−c1u0u0x−c2(t)u02u0x +f(x,t,u0,u0x,u0t)=−c2(t)u02u0x+f(x,t,u0,u0x,u0t),

(18) p2:L(u2)=−c1u0u1x−c1u1u0x−c2(t)u02u1x+F(u0,u1),

where $F\left(u_{0},u_{1}\right)=\left(\partial /\partial p\right)f\left(x,t,\tilde{u},{\tilde{u}}_{x},{\tilde{u}}_{t}\right){|}_{p=0}$.

From (16) we have

(19) u0(x,t)=v1(x,t).

By using the Fourier transform, one can obtain the solution of (17) with the initial condition *u*_1_|_*t*=0_ = 0 as follows:

(20) u1(x,t) =12π∫0t∫−∞+∞[−c2(τ)u02u0ξ+f(ξ,τ,u0,(u0)ξ,(u0)τ)]    ×∫−∞+∞cos⁡[−λ3c3(t−τ)+λ(x−ξ)]dx dξ dτ.

Similarly, one also finds the solution of (18) with the initial condition *u*_2_|_*t*=0_ = 0

(21) u2(x,t)=12π∫0t∫−∞+∞(−c1u0u1x−c1u1u0x−c2(t)u02u1x+F(u0,u1))   ×∫−∞+∞cos⁡[−λ3c3(t−τ)+λ(x−ξ)]dx dξ dτ.

From (11), (12), (13), (20), and (21), the two degree approximate solutions of (5) can be obtained

(22) u2∗(x,t) =c0−6k2c3m2sn(ξ,m)+12k2c3(m2−1)cn(ξ,m)c1(sn(ξ,m)+cn(ξ,m)+dn(ξ,m))  +3k2c3m4sn2(ξ,m)−12k2c3cn2(ξ,m)c1(sn(ξ,m)+cn(ξ,m)+dn(ξ,m))2  +12π∫0t∫−∞+∞(−c2(τ)u02u0ξ+f(ξ,τ,u0,(u0)ξ,(u0)τ)        −c1u0u1x−c1u1u0x        −c2(t)u02u1x+F(u0,u1))    ×∫−∞+∞cos⁡[−λ3c3(t−τ)+λ(x−ξ)]dx dξ dτ,

where *ξ* = *kx* + [−*c*_0_ *kc*_1_ + *k* ^3^ *c*_3_(4*m* ^2^ − 5)]*t* + *ξ*_0_, *k*, *c*_0_, *ξ*_0_ is any constant, *m* is the module, and 0 ≤ *m* ≤ 1, $F\left(u_{0},u_{1}\right)=\left(\partial /\partial p\right)f\left(x,t,\tilde{u},{\tilde{u}}_{x},{\tilde{u}}_{t}\right){|}_{p=0}$,

(23) u0=c0−6k2c3m2sn(ξ,m)+12k2c3(m2−1)cn(ξ,m)c1(sn(ξ,m)+cn(ξ,m)+dn(ξ,m))+3k2c3m4sn2(ξ,m)−12k2c3cn2(ξ,m)c1(sn(ξ,m)+cn(ξ,m)+dn(ξ,m))2,u1=12π∫0t∫−∞+∞[−c2(τ)u02u0ξ+f(ξ,τ,u0,(u0)ξ,(u0)τ)]  ×∫−∞+∞cos⁡[−λ3c3(t−τ)+λ(x−ξ)]dx dξ dτ.

When *m* → 1 and *m* → 0, *u*_2_*(*x*, *t*) degenerates to the following approximate solutions:

(24) u2.1∗(x,t) =c0−6k2c3tanhξc1(tanhξ+2 sec hξ)  +3k2c3tanh2ξ−12k2c3sec h2ξc1(tanhξ+2 sec hξ)2  +12π∫0t∫−∞+∞(−c2(τ)u02u0ξ       +f(ξ,τ,u0,(u0)ξ,(u0)τ)       −c1u0u1x−c1u1u0x       −c2(t)u02u1x+F(u0,u1))    ×∫−∞+∞cos⁡[−λ3c3(t−τ)+λ(x−ξ)]dx dξ dτ,u2.2∗(x,t) =c0+−6k2c3sinξ+12k2c3cos⁡ξc1(sinξ+cos⁡ξ+1)−12k2c3cos⁡2ξc1(sinξ+cos⁡ξ+1)2  +12π∫0t∫−∞+∞(−c2(τ)u02u0ξ        +f(ξ,τ,u0,(u0)ξ,(u0)τ)        −c1u0u1x−c1u1u0x        −c2(t)u02u1x+F(u0,u1))      ×∫−∞+∞cos⁡[−λ3c3(t−τ)+λ(x−ξ)]dx dξ dτ.

By comparing the higher power coefficients of *p*, more higher power approximate solutions can also be obtained, and hence the approximate solution of (1) is found.

## 4. Example 

If *f* = *εu* ^*n*^ is the disturbance term of (5), where 0 < *ε* ≪ 1, (5) becomes

(25) ut+c1uux+c2(t)u2ux+c3u3x=εun.

By using the above method in Section 3, one can find each order approximate solution of elliptic function form for (25) as follows:

(26) u0∗(x,t) =c0−6k2c3m2sn(ξ,m)+12k2c3(m2−1)cn(ξ,m)c1(sn(ξ,m)+cn(ξ,m)+dn(ξ,m))  +3k2c3m4sn2(ξ,m)−12k2c3cn2(ξ,m)c1(sn(ξ,m)+cn(ξ,m)+dn(ξ,m))2,u1∗(x,t) =c0−6k2c3m2sn(ξ,m)+12k2c3(m2−1)cn(ξ,m)c1(sn(ξ,m)+cn(ξ,m)+dn(ξ,m))  +3k2c3m4sn2(ξ,m)−12k2c3cn2(ξ,m)c1(sn(ξ,m)+cn(ξ,m)+dn(ξ,m))2  +12π∫0t∫−∞+∞(−c2(τ)u02u0ξ+εu0n)      ×∫−∞+∞cos⁡[−λ3c3(t−τ)+λ(x−ξ)]dx dξ dτ,u2∗(x,t) =c0−6k2c3m2sn(ξ,m)+12k2c3(m2−1)cn(ξ,m)c1(sn(ξ,m)+cn(ξ,m)+dn(ξ,m))  +3k2c3m4sn2(ξ,m)−12k2c3cn2(ξ,m)c1(sn(ξ,m)+cn(ξ,m)+dn(ξ,m))2  +12π∫0t∫−∞+∞(−c2(τ)u02u0ξ+εu0n−c1u0u1x       −c1u1u0x−c2(t)u02u1x+εnu0n−1)       ×∫−∞+∞cos⁡[−λ3c3(t−τ)+λ(x−ξ)]dx dξ dτ,

where *ξ* = *kx* + [−*c*_0_ *kc*_1_ + *k* ^3^ *c*_3_(4*m* ^2^ − 5)]*t* + *ξ*_0_, *k*, *c*_0_, *ξ*_0_ is any constant, *m* is the module, and 0 ≤ *m* ≤ 1, *u*_0_ = *u*_0_*(*x*, *t*),

(27) u1=12π∫0t∫−∞+∞[−c2(τ)u02u0ξ+εu0n]  ×∫−∞+∞cos⁡[−λ3c3(t−τ)+λ(x−ξ)]dx dξ dτ.

## 5. Conclusion

This work studies the perturbed CKdV equation with variable coefficients by using the homotopic mapping method, and two degree approximate solution of the Jacobi elliptic function form are obtained, which can degenerate to solitary wave approximate solution and trigonometric function approximate solution in the limit cases. Furthermore, the approximate solution of the perturbed CKdV is also obtained. Our results show that the homotopic mapping method is applicable to the variable soliton equations. How to apply this method to high degree and high dimension system remains to be further studied.
